# Cigarette smoke downregulates HDAC2 in rheumatoid arthritis synovial fibroblasts

**DOI:** 10.1186/ar3612

**Published:** 2012-02-09

**Authors:** Anna Engler, Astrid Jüngel, Christoph Kolling, Beat A Michel, Renate Gay, Steffen Gay, Caroline Ospelt

**Affiliations:** 1 Center of Experimental Rheumatology, University Hospital Zurich and Zurich Center of Integrative Human Physiology (ZIHP), Zurich, Switzerland; 2 Schulthess Clinic, Zurich, Switzerland

## Background

Cigarette smoking has been shown as major environmental risk factor for rheumatoid arthritis (RA). Epidemiological studies indicate an association of cigarette smoking with development of RA [1,2], although molecular mechanisms remain unknown. The aim of this study is to analyze the influence of cigarette smoke on the gene expression regulated by histone deacetylases (HDACs) in RA synovial fibroblasts (RASF).

## Methods

RASF obtained from patients undergoing joint replacement surgery were stimulated with freshly prepared cigarette smoke extract (CSE) for 24 hours. Expression of HDACs was measured at the mRNA level by Real-time TaqMan and SYBR green PCR and at the protein level by immunoblot analysis. Global histone 3 (H3) acetylation was analyzed by immunoblot.

## Results

Stimulation of RASF (n = 8-10) with CSE significantly enhanced the expression of HDAC1 (x-fold: 2.0 ± 0.4; p = 0.04), HDAC2 (1.9 ± 0.3; p = 0.02) and HDAC3 (2.4 ± 0.4; p = 0.01) at the mRNA level while the expression of HDAC 4-11 remained unchanged. On the protein level, expression of HDAC1 and HDAC3 were not altered, whereas the expression of HDAC2 protein was decreased in CSE stimulated RASF. No measurable changes in global acetylation of H3 were induced by CSE in RASF (n = 6).

## Conclusion

CSE specifically downregulates the expression of HDAC2 in RASF. Differential regulation of HDAC2 at the mRNA and protein level points to post-transcriptional degradation mechanisms induced by smoking. Even though global H3 acetylation was not changed by CSE, decreased HDAC2 levels might be associated with hyper-acetylation and thus increased expression of specific HDAC2 regulated genes.
